# Shaping triple-conducting semiconductor BaCo_0.4_Fe_0.4_Zr_0.1_Y_0.1_O_3-δ_ into an electrolyte for low-temperature solid oxide fuel cells

**DOI:** 10.1038/s41467-019-09532-z

**Published:** 2019-04-12

**Authors:** Chen Xia, Youquan Mi, Baoyuan Wang, Bin Lin, Gang Chen, Bin Zhu

**Affiliations:** 1 Key Laboratory of Ferro and Piezoelectric Materials and Devices of Hubei Province, Faculty of Physics and Electronic Science, Hubei University, 430062 Wuhan, Hubei China; 2 Faculty of Materials Science and Chemistry, China University of Geosciences, 430074 Wuhan, China; 3 Department of Energy Technology, KTH Royal Institute of Technology, SE-10044 Stockholm, Sweden; 4 School of Materials and Energy, University of Electronic Science and Technology of China, 611731 Chengdu, China; 5 Liaoning Key Laboratory for Metallurgical Sensor and Technology, Northeastern University, 110819 Shenyang, China; 6 Department of Aeronautical and Automotive Engineering, Loughborough University, Ashby Road, Loughborough, LE11 3TU UK

## Abstract

Interest in low-temperature operation of solid oxide fuel cells is growing. Recent advances in perovskite phases have resulted in an efficient H^+^/O^2-^/e^-^ triple-conducting electrode BaCo_0.4_Fe_0.4_Zr_0.1_Y_0.1_O_3-δ_ for low-temperature fuel cells. Here, we further develop BaCo_0.4_Fe_0.4_Zr_0.1_Y_0.1_O_3-δ_ for electrolyte applications by taking advantage of its high ionic conduction while suppressing its electronic conduction through constructing a BaCo_0.4_Fe_0.4_Zr_0.1_Y_0.1_O_3-δ_-ZnO p-n heterostructure. With this approach, it has been demonstrated that BaCo_0.4_Fe_0.4_Zr_0.1_Y_0.1_O_3-δ_ can be applied in a fuel cell with good electrolyte functionality, achieving attractive ionic conductivity and cell performance. Further investigation confirms the hybrid H^+^/O^2-^ conducting capability of BaCo_0.4_Fe_0.4_Zr_0.1_Y_0.1_O_3-δ_-ZnO. An energy band alignment mechanism based on a p-n heterojunction is proposed to explain the suppression of electronic conductivity and promotion of ionic conductivity in the heterostructure. Our findings demonstrate that BaCo_0.4_Fe_0.4_Zr_0.1_Y_0.1_O_3-δ_ is not only a good electrode but also a highly promising electrolyte. The approach reveals insight for developing advanced low-temperature solid oxide fuel cell electrolytes.

## Abstract (web summary)

Solid oxide fuel cells enable efficient electricity generation at high temperatures. Here the authors incorporate a mixed ion-electron semiconductor into another semiconductor to form a p-n junction to suppress electron conduction and enhance ion conduction, leading to a low-temperature electrolyte.

## Introduction

Solid oxide fuel cells (SOFCs) enable highly efficient electric-power generation from a wide variety of fuels. Due to these features of high efficiency, low pollution and fuel flexibility, SOFC technology is widely regarded as a promising solution for addressing the growing global energy demand and the inextricable influence to climate change^1,2^. To date, practical industrial applications of SOFCs have not been successfully realized primarily due to high operational temperatures (over 700 °C) required by the predominant oxygen ion (O^2-^) electrolyte yttrium stabilized zirconia (YSZ), which results in high costs, performance degradation, slow start-up and shut-down cycles, as well as technological complexities^3,4^. Therefore, in recent years, massive efforts have been dedicated worldwide to addressing electrolyte challenges, so that the working conditions of SOFCs can be brought down to a low-temperature (LT) range, particularly 300–600 °C, which enables cheap interconnects and seal, long-term fuel cell lifespan, rapid start-up and shut-down, and broadened application fields^3^. For instance, a great deal of studies have focused on developing ultra-thin film electrolyte layers of YSZ, doped-ceria such as Sm_0.2_Ce_0.8_O_2-δ_ (SDC), and YSZ/Gd_0.1_Ce_0.9_O_2-δ_ (GDC) film on micromachined silicon for micro-SOFCs (μSOFCs), in order to minimize the device ohmic loss and maintain desirable power output at reduced temperatures^5–13^. However, the thin film techniques are confronted by key issues that include high cost, long production period, difficulties on scaling up and μSOFC silicon technology, while doped-ceria is subject to its well-known problem of reduction in hydrogen atmosphere, making them difficult to realize practical LT-SOFCs.

Recent advances in perovskite oxides have led to a new promising strategy for enabling LT-SOFCs by using proton (H^+^) conducting perovskites. For instance, operation of a ceramic fuel cell using a typical proton electrolyte BaCe_0.7_Zr_0.1_Y_0.1_Yb_0.1_O_3-δ_ (BCZYY) and a triple charge (H^+^/O^2−^/e^−^) conducting cathode BaCo_0.4_Fe_0.4_Zr_0.1_Y_0.1_O_3-δ_ (BCFZY) was successfully demonstrated at low temperatures, exhibiting a considerable power output of 445 mW cm^−2^ at 500 °C and possible operation at 350 °C^14^. As a doped derivative of H^+^/O^2−^ conducting BaZr_x_Y_1−x_O_3-δ_ (BZY), the cathode BCFZY gains remarkably activated electron-hole conductivity via heavy B-site doping with Co and Fe while maintaining its high ionic conductivity, leading to good catalytic activity and triple conduction^14,15^. In another featured breakthrough study with respect to protonic perovskite materials, a nickelate SmNiO_3_ (SNO) possessing high initial ionic and electronic conductivity can be utilized as an electrolyte in a LT-SOFC, revealing a peak power output of 225 mW cm^−2^ at 500 °C along with sufficient open-circuit voltage (OCV) of 1.03 V^16^. The SNO electrolyte presented a high H^+^ conductivity at 300–500 °C that is comparable to the best-performing solid electrolytes while its electronic conduction was suppressed via a filling-controlled Mott transition^16^. These interesting studies indicate that perovskite oxides involving H^+^ conduction are more capable at lower temperatures (350–550 °C) compared to conventional SOFCelectrolytes. This is due to the fact that H^+^ conduction in perovskite oxides has a smaller activation energy (*E*_a_) compared to mobile O^2−^ owing to its extremely small ionic size^16,17^. Besides, it has been reported that H^+^ can transport not only in the perovskite structure but also along interfaces of composite materials^18^.

Notably, in spite of the advantage at low temperatures, common proton conducting perovskite electrolytes are unfortunately facing a key challenge of insufficient ionic conductivity, which is constrained by their high grain boundary resistances^19–21^. Over the previous few years, representative proton conductors such as doped barium cerates (BaCe_1−x_M_x_O_3−δ_) generally displayed low proton conductivity of 10^–2^ S cm^-1^ at temperatures below 600 °C^22^. Though some efforts have been dedicated to developing proton-conducting electrolytes by using advanced thin film procedures, current proton conductor fuel cell (PCFC) performance still lags far behind SOFC performance. But on the bright side, proton conducting properties of perovskites, in particular BCFZY and BZY, and their potential versatility are still extremely attractive for enabling low-temperature operation of fuel cells^14,23^. Additionally, the semiconducting nature of perovskite oxides is of great significance, as semiconductors have been frequently applied to explore high-performance electrolyte membranes for LT-SOFCs in recent years^16,24^. If we can utilize these advantages to design new highly ion-conducting electrolytes, perovskite oxides will find greater promise for practical LT-SOFCs.

Following this line and enlightened by above studies, we further promote the promising triple-conducting BZFCY towards application as an electrolyte for LT-SOFCs. A new heterostructure material is designed by incorporating p-type BCFZY with n-type semiconductor ZnO to form a homogeneous composite, which is then sandwiched between two Ni_0.8_Co_0.15_Al_0.05_LiO_2-δ_ (NCAL) electrodes to construct fuel cell devices. The developed heterostructure exhibits appreciable ionic conductivity and fuel cell performance at low operational temperatures of 400–500 °C, realizing good electrolyte functionality by using the p-n heterojunction effect for suppression of electronic conductivity and ionic conductivity promotion. The observed behaviors and measured results suggest that the triple-conductive BZFCY can be a promising electrolyte material for LT-SOFCs via a p-n heterojunction effect. Our approach leads to insight from semiconductor and junction perspectives to design advanced electrolytes.

## Results

### Crystal structure and heterostructure

**Fig. 1:**
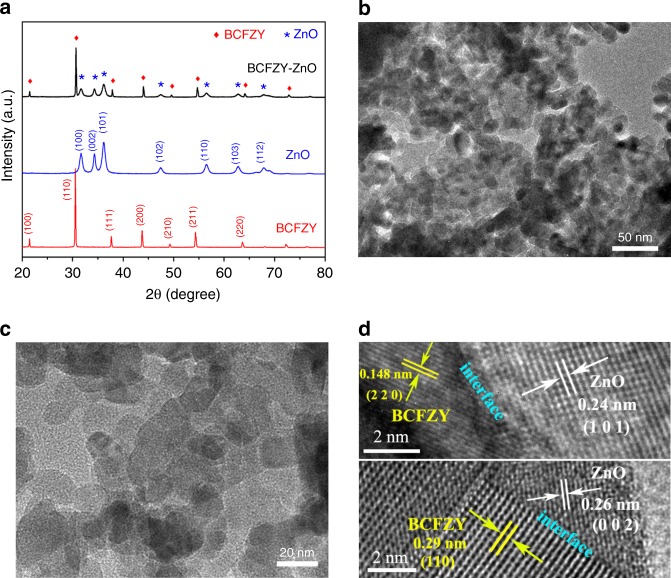
Structure and morphology characterization (**a**) XRD patterns of the prepared BCFZY, commercial ZnO and BCFZY-ZnO composite, (**b**) and (**c**) TEM images of the BCFZY-ZnO composite, (**d**) Two typical HR-TEM images of the BCFZY-ZnO composite showing the interfaces between BCFZY and ZnO

Figure 1a shows the X-ray diffraction (XRD) patterns of as-synthesized BCFZY, BCFZY-ZnO composite and commercial ZnO. The diffraction peaks of BCFZY can be well indexed to a single cubic perovskite structure without any impurity peaks, demonstrating a pure BCFZY phase, while those of ZnO match well to a standard hexagonal wurtzite structure^14,25,26^. In the patterns of BCFZY-ZnO, all diffraction peaks can be assigned to BCFZY and ZnO, evidencing that the two phases co-existed in the composite and no chemical reaction occurred between them. Furthermore, transmission electron microscope (TEM) and scanning electron microscope (SEM) are employed to investigate the morphology and micro-structure of BCFZY-ZnO. Figure 1b, c provide two TEM images of the composite with different amplifications to depict its homogeneous distribution of nano-scale particles. It is clearly seen that plenty of contacts formed between these particles or grains, as also shown in Supplementary Fig. 1a. Figure 1d further gives two typical high-resolution TEM (HR-TEM) images of the BCFZY-ZnO heterostructure, in both of which, the presence of BCFZY and ZnO are clearly observed. In the upper image of Fig. 1d, it can be seen the well-defined crystalline fringes with lattice spacings of 0.148 and 0.24 nm corresponding to the (220) crystal plane of BCFZY and the (101) crystal plane of ZnO, respectively, while in the one below, two lattice spacings of 0.29 and 0.26 nm corresponding to the (110) planes of BCFZY and the (002) plane of ZnO are also observed. The heterophasic interfaces between BCFZY and ZnO are therefore clearly identified, as annotated in the images. Such type of interfaces are found to form at either particle level or grain level, more details depicting these microstructure features are in Supplementary Fig. 1b and 1c, separately. The XRD and TEM results thus authenticate the formation of a heterostructure composite for our prepared BCFZY-ZnO.

### Fuel cell activation process and electrochemical performance

Current-voltage (I–V) characteristic tests offer direct verification of the electrolyte functionality of the BCFZY-ZnO composite in LT-SOFC. Our investigation showed that the fuel cell current densities are closely correlated with the mass ratios of BCFZY:ZnO. It was found that BCFZY-ZnO with mass ratio of 2:1 (2BCFZY-ZnO) is the optimal composite among five groups of samples (8:2, 7:3, 2:1, 6:4, 5:5), by referring to their fuel cell power densities as represented in Supplementary Fig. 2. To achieve the desired fuel cell performance, 2BCFZY-ZnO is assigned as our study case hereinafter.

**Fig. 2:**
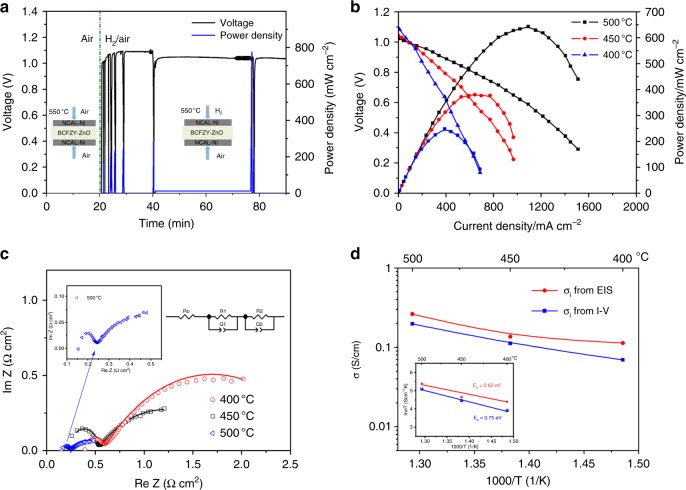
Electrochemical performance and conductivity. **a** Activation process for 7 cycles of the BCFZY-ZnO electrolyte-based SOFC at 550 °C to achieve active voltage and current, (**b**) Electrochemical performance of the stabilized BCFZY-ZnO electrolyte based SOFC at 400–500 °C, (**c**) Impedance spectra of BCFZY-ZnO fuel cell measured in H_2_/air at 400, 450, and 500 °C, and the corresponding equivalent circuit model for fitting, (**d**) Ionic conductivity and activation energy plots of BCFZY-ZnO as a function of 1000/T obtained from EIS and I-V curve at 400–500 °C

Activation process is of great practical relevance to evaluate the electrode and electrolyte performance. Figure 2a shows the recorded activation process of a BCFZY-ZnO electrolyte based SOFC at 550 °C. After supplying H_2_/air, initial activation took place at anode and cathode regions when H_2_ is catalyzed to form protons and electrons, and cathodic surface provided electrons to O_2_ to form oxide ions, this synchronously gave rise to a high potential difference. Observed is the rapid response of increasing voltage in this initial fuel cell activation process, as shown in Fig. 2a, reflecting the high catalytic activity of electrodes. Subsequently, the cell was activated by repeatedly operating the cell (7 cycles) in order to continuously inject sufficient protons into the electrolyte, thus facilitating the proton conductivity and leading to enhancive cell current outputs. This process is necessary for the developed fuel cell because there were no intrinsic protons in the used electrolyte material. Meanwhile, the electrode could be also activated by current passage when operated the cell. Eventually, the cell voltage approached a stable value of 1.01 V, and the corresponding power output was improved from initial 162 mW cm^−2^ up to 775 mW cm^−2^. The high voltage and power output thus confirm the feasibility of BCZYY as a competent electrolyte material in LT-SOFC, indicating that BCZYY holds great potential as not only electrode but also electrolyte.

After the fuel cell was fully activated and stabilized, its electrochemical performance was measured in the temperature range of 400–500 °C. Figure 2b presents the I–V characteristics and power density curves of a cell using hydrogen as a fuel and air as an oxidant. The cell shows a superior OCV of 1.08, 1.03, and 1.01 V at 400, 450, and 500 °C, respectively, excluding a possibility of short circuit although semiconductors were used in the electrolyte layer. The stabilized fuel cell delivers attractive peak power output of 248–643 mW cm^-2^ at 400–500 °C, which is comparable or even higher than those of the SOFCs using ceramic YSZ, SDC and BCZYY electrolytes under the identical operating conditions^14,27,28^. Such low-temperature performance is also superior to the reported results of a few μSOFCs with thin-film YSZ electrolytes, which, for example, exhibit 450 mW cm^−2^ at 500 °C and 155 mW cm^−2^ at 510 °C^9,10^. However, when compared to the advanced level of thin-film YSZ μSOFCs (1037 mW cm^−2^ at 500 °C) and YSZ/GDC μSOFCs (1175 mW cm^−2^ at 520 °C)^6,8,11,12^, the studied BCFZY-ZnO fuel cells show substantially lower power outputs, although this comparison is regardless of the technological differences between the macro-and micro-scale SOFCs. Above results imply that on the one hand, the adopted approach has successfully presented a promising technology of heterostructure electrolyte based LT-SOFC; on the other hand, much more efforts, especially engineering efforts such as thin film techniques are in desperate demand to facilitate it to a more advanced technology.

### Impedance spectra analysis and ionic conductivity

The electrical properties of BCFZY and BCFZY-ZnO with respect to temperature were studied by electrochemical impedance spectra (EIS). Supplementary Fig. 3 shows the EIS results acquired in air at 500 ^o^C. The EIS curve of BCFZY shows a spot in the real Z-axis, indicating that BCFZY presents dominating intrinsic electron-hole conduction in air, while the EIS of BCFZY-ZnO in air displays an ionic conductor behavior^29^. This implies the composite gained remarkably enhanced ionic conduction and suppressed electronic conduction after the introduction of ZnO to form a heterostructure. Figure 2c exhibits the representative impedance spectra of BCFZY-ZnO fuel cell measured in H_2_/air at 400, 450, and 500 °C. For comparison, the impedance spectra obtained at lower temperatures of 300 and 350 °C are also shown in Supplementary Fig. 4. These experimental EIS data are fitted with an equivalent circuit of R_o_(R_1_Q_1_)(R_2_Q_2_) (inset in Fig. 2c), and the simulated parameters are summarized in Supplementary Table 1. As a result, the small values for the sum of ohmic and grain-boundary resistances manifest the high conductivity of BCFZY-ZnO at 400–500 °C. The electrode polarization resistance also exhibits small values of 0.36–2.04 Ω cm^2^ over this temperature range, suggesting a high electrode reaction activity. This should be owed to the cathode functionality of BCFZY that contributed to the electrolyte/cathode region, which leads to fast oxygen reduction reaction (ORR) kinetics at the BZFCY-ZnO/NCAL interface^14,15^.

According to the simulated R_o_ + R_1_ at various temperatures, the ionic conductivity (σ_i_) of BCFZY-ZnO is calculated, as shown in Fig. 2d. In addition, the σ_i_ results can also be obtained from the slope of polarization curve (in Fig. 2b) at the ohmic polarization region, as also plotted in Fig. 2d^30^. The σ_i_ acquired by EIS reveals fairly high values of 0.11–0.26 S cm^-1^ at 400–500 °C, while the σ_i_ obtained from I-V curve ranges from 0.07 to 0.20 S cm^-1^ in the same temperature range. The attained ionic conductivity is remarkably higher than the reported values of predominant oxygen-ion conductors (SDC ~0.05 S cm^−1^ at 700 °C, GDC 0.04 S cm^−1^ at 700 °C, ceramic YSZ 0.13 S cm^-1^ at 1000 °C, GDC/YSZ mixture film ~0.01 S cm^−1^ at 1000 °C, large-scale thin-film YSZ 0.005 S cm^-1^ at 500 °C), and also superior to that of typical proton conductor BaCe_0.7_Zr_0.1_Y_0.2_O_3-δ_ (BCZY 0.02 S cm^−1^ at 700 °C)^13,31–35^. It is believed such favorable σ_i_ is a combined result of the high H^+^/O^2−^ conductivity inherited from BCFZY along with the composite interface effect of BCFZY-ZnO. Moreover, in this ionic transport case, the thermally activated behavior of ions is obeyed according to Arrhenius equation σT = Aexp[−*E*_a_/(kT)], thus the Arrhenius representation of σ_i_ for BZFCY-ZnO is obtained as shown in Fig. 2d inset. The calculated activation energy *E*_a_ are 0.62 and 0.75 eV corresponding to the σ_i_ from EIS and I–V, respectively, which are lower than the reported results of oxygen-ion conductors^36^. Take into account that BZFCY possesses appreciable H^+^ conduction, which requires lower *E*_a_ compared to O^2−^, it is deducible the BZFCY-ZnO composite continued the H^+^ conducting property and thus gained a low *E*_a_.

### Hybrid H^+^/O^2−^ conduction of the composite

Above study shows that the prepared BZFCY-ZnO composite has underlying proton conduction inherited from BCFZY, leading to a hybrid H^+^/O^2−^ conducting property and the extraordinarily high level of ionic conductivity at 400–500 °C. To further verify the proton-conducting property of BZFCY-ZnO, an O^2−^/e^−^ blocking fuel cell in configuration of *NCAL-Ni/BCZY/BZFCY-ZnO/BCZY/NCAL-Ni* was fabricated by using proton conductor BCZY as the filtering layer (or blocking layer). This blocking cell approach has been reported in previous studies to measure a specific ionic conductivity via getting rid of the influence of other carriers^37,38^. The I-V characteristics for the assembled blocking cell were tested under the identical condition as above. Since that BCZY has high H^+^ but negligible O^2−^/e^−^ conduction, the BCZY/BZFCY-ZnO/BCZY trilayer electrolyte would only allow H^+^ to pass through while filtering out O^2−^ and e^−^^34,39^. In this case, only H^+^ contributes to the fuel cell current output, and thus the proton conductivity of BZFCY-ZnO can be determined from the I–V polarization curve.

**Fig. 3:**
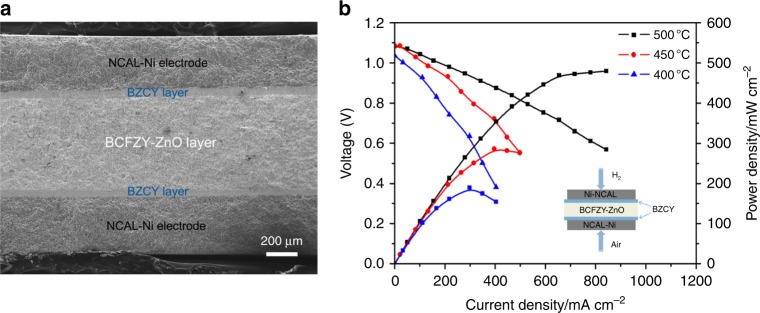
Hybrid H^+^/O^2-^ conduction (**a**) A cross-sectional SEM image of the O^2-^/e^-^ blocking cell in configuration of NCAL-Ni/BCZY/BZFCY-ZnO/BCZY/NCAL-Ni, (**b**) I–V characteristics of the cell measured at 400–500 °C

Figure 3a displays the cross-sectional view of the cell examined by SEM. Five layers corresponding to NCAL electrodes, thin BCZY filters and BZFCY-ZnO electrolyte are clearly distinguished in the SEM image, indicating the successful construction of O^2−^/e^−^ blocking cell. Two enlarged SEM images for the same cross-section are depicted in Supplementary Fig. 5, which show the thickness (700 µm) of the trilayer and dense electrolyte along with porous electrode of the cell, respectively. Figure 3b shows the typical I–V characteristics of the O^2−^/e^−^ blocking fuel cell measured at 400–500 °C after activation and stabilization. The fuel cell demonstrated power outputs of 189–479 mW cm^−2^ and OCVs of 1.03–1.09 V over this temperature range. The current output confirms the considerable proton conducting property of BZFCY-ZnO. Based on the polarization curve, the proton conductivity of BZFCY-ZnO can be estimated, exhibiting appreciable values of 0.039–0.098 S cm^−1^ at 400–500 °C. This should be owed to the activation process of our fuel cell that injects protons into the BZFCY-ZnO layer as described earlier. The obtained proton conductivity fills approximately half portion of the total ionic conductivity, as plotted in Supplementary Fig. 6, indicative of the hybrid H^+^/O^2−^ conduction of the composite.

It should be clarified that in the blocking cell, more ohmic and interfacial polarization losses can be induced due to the intercalary filtering layers, fundamentally limiting the current output and cell performance. As a consequence, the derived proton conductivity from polarization curve is always lower with respect to the actual values. In our study, BCZY was cautiously picked as the filtering layer due to the fact that it possesses proximate high proton conduction, compatible coefficient of thermal expansion, and good lattice matching with BCFZY, which are anticipated to lead to minimal ohmic and interfacial polarization loss to the cell. In this regard, the blocking cell is feasible and effective to confirm the proton conduction and estimate the conductivity with less error.

### Electron subtraction and ion enhancement by heterojunction

From a traditional point of view towards electrolytes, semiconductor materials with electronic conduction are unfavorable for realizing a normal operation of SOFCs^40^. However, our findings reveal it is feasible to demonstrate BCFZY-ZnO electrolyte in SOFC with excellent ionic conductivity, high OCVs and power densities at low temperature range of 400–500 °C. This has been realized by incorporating the p-type BCFZY with a n-type semiconductor ZnO to form a heterostructure composite. It has been shown by HR-TEM characterization that a fine heterostructure with massive interfacial contacts between BCFZY and ZnO were established in the composite. By considering the conduction types of the two materials, we infer that the successful demonstration is due to a p-n heterojunction effect when charge redistribution occurred at the particle and grain interfaces between p-BCFZY and n-ZnO, inducing gradient energy band and built-in electric field at the interface region. That is conducive to separating electrons and holes to avoid short circuit, and simultaneously boosting the interfacial ionic transportation^41,42^. In this way, high fuel cell OCV and electrochemical performance can be guaranteed.

**Fig. 4:**
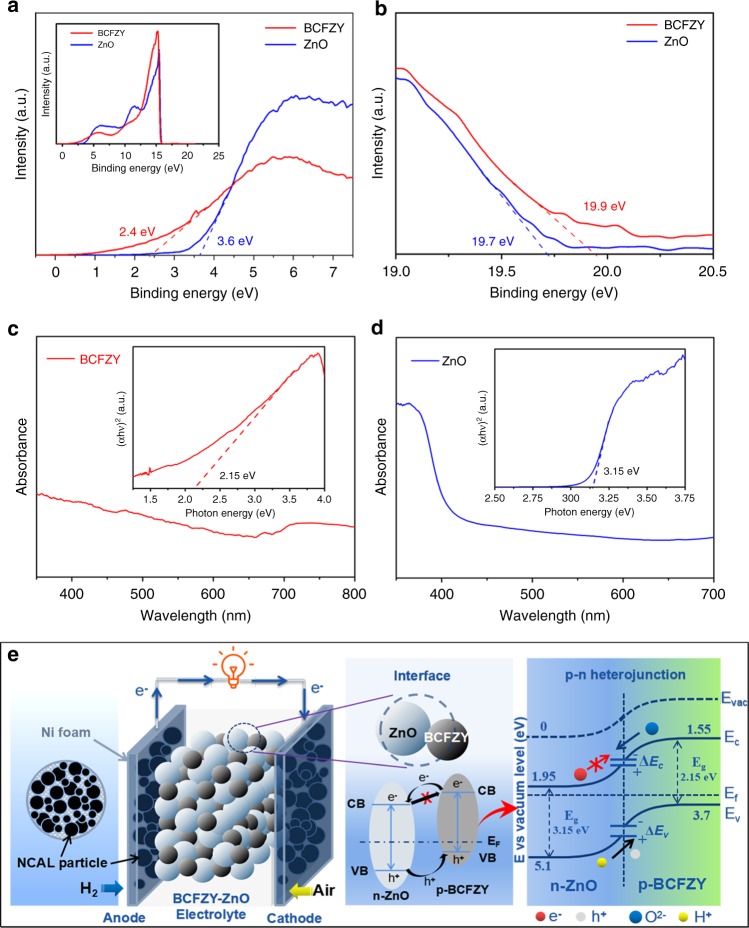
Absorption spectra and schemetic for the mechanism. **a** UPS plots of BCFZY and ZnO with magnified views of the low-binding-energy cutoff, and **b** the high-binding-energy cutoff region. UV-vis absorption spectra and the calculated bandgap of **c** BCFZY and **d** ZnO. **e** Schematic diagram of a typical p-n heterojunction formed at the heterophasic interface of the BCFZY-ZnO electrolyte layer and the corresponding energy band alignment mechanism proposed for interpreting the charge separation and ionic transportation process

To verify the conjecture, ascertaining semiconductor type and energy band level of the materials are pivotal. Hall-effect measurements of BZFCY and ZnO were firstly performed to examine the material conduction type. The parameters including hall coefficient, hall mobility, carrier type, and carrier concentration are listed in Supplementary Table 2, manifesting that BZFCY is p-type semiconductor in air circumstance and n-type semiconductor in reducing condition, while ZnO maintains its well-known n-type conduction (in air) character in reducing conditions^43^. Thus, in fuel cell operational conditions the p-type BCFZY can build up a p-n junction with n-type ZnO. In order to investigate the p-n heterojunction, ultraviolet photoelectron spectra (UPS) and UV-vis absorption spectra of BCFZY and ZnO were measured to obtain their valence band (VB) maxima and energy bandgaps, as shown in Fig. 4a–d. In UPS spectra, the energy is calibrated with respect to He-I photo energy of 21.21 eV, determining the valence band maxima via defining the low-binding and high-binding energy cutoff. Based on UV-vis spectra, bandgap can be obtained by using the Kubelka-Munk function. It is acquired from UPS and UV-vis results that the VB levels of BCFZY and ZnO are 3.7 and 5.1 eV, and their bandgaps are 2.15 and 3.15 eV, respectively. On basis of which, the corresponding conduction band (CB) levels can be determined as 1.55 eV for BCFZY and 1.95 eV for ZnO. These parameters indicate that the p-type BCFZY and n-type ZnO can form the desirable p-n heterojunction. In light of the conduction features and energy levels, a possible mechanism is proposed to interpret the charge separation (electron-blocking) process and electrochemical enhancement of our BCFZY-ZnO fuel cell, as presented in Fig. 4e.

Figure 4e schematically illustrates the structures of the studied SOFC device and BCFZY-ZnO composite along with the energy band alignment of BCFZY-ZnO p-n heterojunction. Commonly when p-type and n-type semiconductors are contacted, redistribution of charges at the interface constitutes a space-charge region (depletion layer) with built-in electric field pointing from n-type towards p-type region^44,45^. Since that p-BCFZY and n-ZnO have difference energy band levels, to reach a continuous Fermi energy level throughout the whole junction, conduction band offset (ΔE_c_) and valence band offset (ΔE_v_) were induced and thus formed potential barriers. The electric field was able to prevent the generated and intrinsic electrons from passing through the junction as depicted in Fig. 4e, dedicating to suppressing the high electronic conduction of the two semiconductors. Meanwhile, charged species H^+^ and O^2−^ can be driven by the built-in electrostatic force, gaining accelerated transportation and lowered activation energy^44^. This ionic enhancement process takes place at the space-charge region of p-n junction, which is the interface/grain boundary between BCFZY and ZnO. Thus, the grain-boundary behavior of BCFZY-ZnO could be partially influenced by such interface effect, resulting in significantly reduced grain-boundary resistance as shown in Supplementary Table 1. Moreover, based on this mechanism, the highest power density of 2BCFZY-ZnO cell as compared with other compositions can be easily understood: at the optimal ratio of 2:1, the contents of BCFZY and ZnO are perfectly matched in the composite to form most sufficient interface and p-n heterojunctions. In contrast, the 8BCFZY-2ZnO composite with majority BCFZY but minor ZnO is hard to form sufficient BCFZY-ZnO heterophase interface. In other words, the interfacial pn junction effect is more remarkable in 2BCFZY-ZnO than in other compositions. Owing to these benefits, our designed 2BCFZY-ZnO composite can exhibit excellent ionic conductivity and high fuel cell performance. Besides, of particular note is that the band diagram in Fig. 4e is only valid for the case at room temperature, because the energy levels of BCFZY and ZnO are measured at room temperature. The actual band alignment for the case under SOFC in-situ operating condition will be a subject of future study.

To further show the reliability of our idea and bearings on fuel cell technology, the durability of BCFZY-ZnO SOFCs was assessed at a low operating temperature of 450 °C. Currently achieved results show that the cells can be subjected to 30 h stable operation at a stationary current density (470 mA cm^−2^) in H_2_/air circumstance, with a constant working voltage of 0.61 V retained for the duration of 26 h (Supplementary Fig. 7). For longer-term durability (>200 h), more compatible electrode materials and extensive engineering efforts are required and will be a subject of our follow-up work regarding this technology.

## Discussion

Comparing to latest breakthrough studies of PCFCs reported by Haile’s and Lee’s groups^46,47^, our fuel cell showed similar area-specific ohmic resistance (ASR_o_, 0.16 Ω cm^2^), but significantly smaller area-specific polarization resistance (ASR_p_, 0.36 Ω cm^2^) vs the two references (0.61 and 0.72 Ω cm^2^) at 500 °C, thus demonstrating higher power density (643 mW cm^−2^ vs 548 mW cm^−2^ and 546 mW cm^-2^ at 500 °C). The small ohmic resistance should be a result of the high conductivity of BCFZY-ZnO, which is mainly because of the high interfacial ionic conduction arisen from the p-n junction effect at grain boundaries (heterophasic interfaces). The exceptionally low electrode polarization resistance can be primarily ascribed to the H^+^/O^2−^/e^−^ triple-conduction of the used heterostructure BCFZY-ZnO that contributed to electrolyte/electrode region, benefiting from which, both ORR and hydrogen oxidation reaction (HOR) kinetics of the cell were enhanced. In contrast, other typical proton electrolytes like BCZYY and BCZY have no such kind of feature to facilitate an electrode reaction. Generally, low ASR_p_ accounts for a pivotal part of good cell performance in SOFCs (or PCFCs) especially at low temperatures. Thus, the proposed BCFZY-ZnO heterostructures, integrating advantages of high ionic conduction and good electrode catalytic function, is capable of delivering high fuel cell performance.

The present work highlights that triple-conducting perovskite BCFZY holds great promise as a new type of electrolyte candidate for LT-SOFC. In contrast to SNO, which is based on a Mott transition to suppress its electronic conduction, the BCFZY realizes its electrolyte functionality of electronic insulation via a microcosmic p-n heterojunction effect in the form of a heterostructure composite^16^. More significantly, the established built-in electric field of the junction can promote ionic transport when O^2−^ and H^+^ pass through the space-charge region, thus giving rise to enhanced ionic conductivity and reduced activation energy, more than just sustaining the original ionic conductivity of BCFZY. This approach departs from traditional studies of SOFC electrolytes that primarily rely on cation substitution of insulators^31^; here the strategy is based on semiconductor characteristics and energy band levels to explore and develop advanced electrolytes. In this way, semiconducting SOFC electrode materials will no longer be restricted to their conventional role as merely an electrode, but find new prospect as electrolytes for LT-SOFCs in a form of heterostructure with matched secondary semiconductors.

In summary, a highly promising triple-conducting cathode BCFZY was developed for LT-SOFC electrolyte application by forming a heterostructure composite with ZnO. The prepared BCFZY-ZnO composite exhibited extraordinary ionic (hybrid H^+^/O^2−^) conductivity and good fuel cell performance at low temperatures (power densities of 643 mW cm^−2^ and OCV of 1.01 V at 500 °C), exhibiting an appreciable electrolyte functionality without apparent short-circuiting issue. Consider the semiconductor and micro-structure features, these interesting results can be attributed to a p-n heterojunction effect at the interface between BCFZY and ZnO, which can effectively suppress the electronic conduction and synchronously facilitate the ionic transport. A possible mechanism based on energy band alignment was proposed to illustrate the electronic blocking and ionic enhancement processes. The favorable electrochemical performance suggests that our study has successfully shaped the triple-conducting semiconductor BCFZY into the electrolytes for LT-SOFCs. The p-n heterostructure approach provides a facile strategy to exploit advanced electrolytes for LT-SOFCs from the existing electrodes and semiconductors.

## Methods

### Powder preparation

BaCo_0.4_Fe_0.4_Zr_0.1_Y_0.1_O_3_ (BCFZY) powders were synthesized by a sol-gel method. Ba(NO_3_)_2_ (Sigma Aldrich, 99.5%), Co(NO_3_)_2_·6H_2_O (Sigma Aldrich, 99%), Fe(NO_3_)_3_·6H_2_O (Sigma Aldrich, 99%), Zr(NO_3_)_4_ (Sigma Aldrich, 99.99%), Y(NO_3_)_3_·6H_2_O (Sigma Aldrich, 99%), C_6_H_8_O_7_·H_2_O (Sigma Aldrich, 99.5%), and C_10_H_16_N_2_O_8_ (Sigma Aldrich, 99.5%) were used as precursors in the synthesis. In brief: first, Ba(NO_3_)_2_, Co(NO_3_)_2_·6H_2_O, Fe(NO_3_)_3_·6H_2_O, Zr(NO_3_)_4_, Y(NO_3_)_3_·6H_2_O were dissolved in ethylene diamine tetraacetic acid (EDTA, Sigma Aldrich) according to the stoichiometric ratio under continuous heating and stirring. Then C_6_H_8_O_7_·H_2_O and C_10_H_16_N_2_O_8_ were added to the solution with the mole proportions of metal cation: citric acid: EDTA = 1: 1.5: 1. Afterwards, NH_3_·H_2_O (Sigma Aldrich) was introduced to the solution to adjust the pH to approximately 9, until the solution became transparent. Subsequently, the solution was stirred for 14 h and then continuously stirred and heated for 4 h to evaporate the water, in this way, a gray gel was obtained. Then, the gels were calcined at 1000 °C in air for 5 h to form powder, followed by an adequately grind to obtain our BZFCY sample.

BCFZY-ZnO composites was prepared via a solid mixing procedure, by blending and grinding the resultant BCFZY and commercial ZnO (Sigma Aldrich, 99%) powders in an optimal mass ratios of 2:1. The mixture powders were then sintered at 700 °C for 2 h and ground completely again to obtain BCFZY-ZnO composite sample.

### Fell cell construction

SOFCs with BCFZY-ZnO composite electrolytes were fabricated via a dry pressing procedure, by compacting the BCFZY-ZnO electrolyte powder between two pieces of Ni_0.8_Co_0.15_Al_0.05_LiO^2−^_δ_-pasted Ni foam (NCAL-Ni) electrodes uniaxially under a load of 250 MPa into one pellet, following by applying silver paste onto the electrode surface as the current collector. The assemble SOFC devices are in a structure of NCAL-Ni/BCFZY-ZnO/NCAL-Ni, with a thickness of approximate 1.5 mm and active area of 0.64 cm^2^. NCAL-Ni electrodes were made by mixing NCAL powders with terpineol solvent to get slurry, which was then pasted on Ni foam following by desiccation at 150 °C for 0.5 h to form NCAL-Ni. The O^2-^/e^-^ blocking cell was fabricated in the same way as above by additionally using two blocking layers of BaCe_0.7_Zr_0.1_Y_0.2_O_3-δ_ (BCZY): the powder of trilayer electrolyte BCZY/BCFZY-ZnO/BCZY was dry pressed between two NCAL-Ni electrodes to form an architecture of NCAL-Ni/BCZY/BCFZY-ZnO/BCZY/NCAL-Ni with the same active area and thickness as above fuel cells. All these fuel cells experienced an online sintering at 600 °C for 0.5 h prior to operation and performance measurement.

### Material characterization

The crystal structures of BCFZY, ZnO and BCFZY-ZnO were carried out by Bruker D8 Advanced X-ray diffractometer (XRD) with Cu Kα (λ = 1.54060 Å) source, tube voltage of 45 kV, and tube current of 40 mA. The diffraction patterns were collected in the 2 theta range between 20 and 80^o^ with intervals of 0.02^o^. The morphology of the composite particles and fuel cell cross-section were investigated by a field emission scanning electron microscope (FE-SEM, JEOL JSM7100F field, Germany). Further micro-structure of composite specimen was investigated using a transmission electron microscope (TEM, JEOL JEM-2100F) operating under accelerating voltage of 200 kV. The Hall-effect of BZFCY and ZnO were measured using a Lake Shore 8400 Hall Measurement system to determine their hall coefficient, hall mobility, carrier type, and carrier concentration. The valence band level maximums were attained via ultraviolet photoelectron spectroscopy (UPS) measurements performed with an unfiltered HeI (21.22 eV) gas discharge lamp and a total instrumental energy resolution of 100 meV. The UV-vis absorption spectra of the materials were tested with a UV3600 spectrometer (MIOSTECHPTY Ltd.).

### Electrochemical measurements

The electrical and electrochemical properties of the composites were studied by electrochemical impedance spectra (EIS) performed with a Gamry Reference 3000 electrochemical work station (Gamry Instruments, USA). The data were collected under open circuit voltage (OCV) modes and the applied frequency was in the range of 0.1–10^5^ Hz with an AC voltage signal of 10 mV in amplitude. The fuel cell current-voltage characteristics and power densities were measured at 400–550 °C with dry hydrogen and air as fuel and oxidant (120–150 mL min^−1^), respectively. The measurements were performed using a IT8511 electronic load (ITECH Electrical Co., Ltd., China) while the data were recorded by a IT7000 software with scan speed of 0.02 A s^−1^ in the current-voltage sweep.

## Supplementary information

Supplementary Info

## Data Availability

The data that support the findings of this study are available from the corresponding author on request.

## References

[1] Ormerod RM (2003). Solid oxide fuel cells. Chem. Soc. Rev..

[2] Singhal SC (2002). Solid oxide fuel cells for stationary, mobile, and military applications. Solid State Ion..

[3] Wachsman ED, Lee KT (2011). Lowering the temperature of solid oxide fuel cells. Science.

[4] Goodenough JB (2000). Ceramic technology: oxide-ion conductors by design. Nature.

[5] Chen YY, Wei WCJ (2006). Processing and characterization of ultra-thin yttria-stabilized zirconia (YSZ) electrolytic films for SOFC. Solid State Ion..

[6] Huang H (2007). High-performance ultrathin solid oxide fuel cells for low-temperature operation. J. Electrochem. Soc..

[7] Shim JH, Chao CC, Huang H, Prin FB (2007). Atomic layer deposition of yttria-stabilized zirconia for solid oxide fuel cells. Chem. Mater..

[8] Kerman K, Lai BK, Ramanathan S (2012). Nanoscale Compositionally Graded Thin‐Film Electrolyte Membranes for Low‐Temperature Solid Oxide. Fuel Cells Adv. Energy Mater..

[9] Takagi Y, Lai BK, Kerman K, Ramanathan S (2011). Low temperature thin film solid oxide fuel cells with nanoporous ruthenium anodes for direct methane operation. Energy Environ. Sci..

[10] Tsuchiya M, Lai BK, Ramanathan S (2011). Scalable nanostructured membranes for solid-oxide fuel cells. Nat. Nanotechnol..

[11] Kerman K, Lai BK, Ramanathan S (2011). Pt/Y0. 16Zr0. 84O1. 92/Pt thin film solid oxide fuel cells: Electrode microstructure and stability considerations. J. Power Sources.

[12] Su PC, Chao CC, Shim JH, Fasching R, Prinz FB (2008). Solid oxide fuel cell with corrugated thin film electrolyte. Nano. Lett..

[13] Garbayo I (2014). Full ceramic micro solid oxide fuel cells: towards more reliable MEMS power generators operating at high temperatures. Energy Environ. Sci..

[14] Duan C (2015). Readily processed protonic ceramic fuel cells with high performance at low temperatures. Science.

[15] He W (2017). BaCo_0.7_Fe_0.22_Y_0.08_O_3-δ_ as an active oxygen reduction electrocatalyst for low-temperature solid oxide fuel cells below 600°C. ACS Energy Lett..

[16] Zhou Y (2016). Strongly correlated perovskite fuel cells. Nature.

[17] Yamazaki Y, Hernandez-Sanchez R, Haile SM (2009). High total proton conductivity in large-grained yttrium-doped barium zirconate. Chem. Mater..

[18] Wang X (2011). Ceria-based nanocomposite with simultaneous proton and oxygen ion conductivity for low-temperature solid oxide fuel cells. J. Power Sources.

[19] Souza ECCD, Muccillo R (2010). Properties and applications of perovskite proton conductors. Mater. Res..

[20] Katahira K, Kohchi Y, Shimura T, Iwahara H (2000). Protonic conduction in Zr-substituted BaCeO_3_. Solid State Ion..

[21] Iguchi F, Sata N, Tsurui T, Yugami H (2007). Microstructures and grain boundary conductivity of BaZr_1-x_Y_x_O_3_ (x=0.05, 0.10, 0.15) ceramics. Solid State Ion..

[22] Barison S (2008). Barium Non‐Stoichiometry Role on the Properties of Ba_1+x_Ce_0.65_Zr_0.20_Y_0.15_O_3–δ_ Proton Conductors for IT‐SOFCs. Fuel Cells.

[23] Bae K (2017). Demonstrating the potential of yttrium-doped barium zirconate electrolyte for high-performance fuel cells. Nat. Commun..

[24] Zhu B, Yun S, Lund PD (2018). Semiconductor‐ionic materials could play an important role in advanced fuel‐to‐electricity conversion. Int. J. Energy Res..

[25] Fang S, Wang S, Brinkman KS, Chen F (2014). A sinteractive Ni–BaZr_0.8_Y_0.2_O_3-δ_ composite membrane for hydrogen separation. J. Mater. Chem. A.

[26] Suwanboon S, Amornpitoksuk P, Haidoux A, Tedenac JC (2008). Structural and optical properties of undoped and aluminium doped zinc oxide nanoparticles via precipitation method at low temperature. J. Alloy. Compd..

[27] Saebea D, Authayanun S, Patcharavorachot Y, Chatrattanawet N, Arpornwichanop A (2018). Electrochemical performance assessment of low-temperature solid oxide fuel cell with YSZ-based and SDC-based electrolytes. Int. J. Hydrog. Energy.

[28] Zhao L (2008). Optimization on technical parameters for fabrication of SDC film by screen-printing used as electrolyte in IT-SOFC. J. Phys. Chem. Solids.

[29] Wang B (2016). B. Preparation and characterization of Sm and Ca co-doped ceria–La_0.6_Sr_0.4_Co_0.2_Fe_0.8_O_3-δ_ semiconductor ionic composites for electrolyte-layer-free fuel cells. J. Mater. Chem. A.

[30] Qiao Z (2018). Electrochemical and electrical properties of doped CeO^2−^ZnO composite for low-temperature solid oxide fuel cell applications. J. Power Sources.

[31] Mahato N, Banerjee A, Gupta A, Omar S, Balani K (2015). Progress in material selection for solid oxide fuel cell technology: A review. Prog. Mater. Sci..

[32] Prabhakaran K, Beigh MO, Lakra J, Gokhale NM, Sharma SC (2007). Characteristics of 8 mol% yttria stabilized zirconia powder prepared by spray drying process. J. Mater. Process Technol..

[33] Fu YP, Wen SB, Lu CH (2008). Preparation and characterization of samaria‐doped ceria electrolyte materials for solid oxide fuel cells. J. Am. Ceram. Soc..

[34] Fabbri E, D’Epifanio A, Di Bartolomeo E, Licoccia S, Traversa E (2008). Tailoring the chemical stability of Ba(Ce_0.8-x_Zr_x_)Y_0.2_O_3-δ_ protonic conductors for intermediate temperature solid oxide fuel cells (IT-SOFCs). Solid State Ion..

[35] Zuo C, Zha S, Liu M, Hatano M, Uchiyama M (2006). Ba (Zr_0.1_Ce_0.7_Y_0.2_)O_3–δ_ as an electrolyte for low‐temperature solid‐oxide fuel cells. Adv. Mater..

[36] Wei T (2014). Sr_3−3x_Na_3x_Si_3_O_9−1.5x_(x=0.45) as a Superior Solid Oxide-ion Electrolyte for Intermediate Temperature-Solid Oxide Fuel Cells. Energy Environ. Sci..

[37] Fan B, Yan J, Yan X (2011). The ionic conductivity, thermal expansion behavior, and chemical compatibility of La_0.54_Sr_0.44_Co_0.2_Fe_0.8_O_3-δ_ as SOFC cathode material. Solid State Sci..

[38] Wang B (2016). CoFeZrAl-oxide based composite for advanced solid oxide fuel cells. Electrochem. Commun..

[39] Sun W, Shi Z, Wang Z, Liu W (2015). Bilayered BaZr_0.1_Ce_0.7_Y_0.2_O_3‑δ_/Ce_0.8_Sm_0.2_O_2‑δ_ electrolyte membranes for solid oxide fuel cells with high open circuit voltages. J. Membr. Sci..

[40] Kharton VV, Marques FMB, Atkinson A (2004). Transport properties of solid oxide electrolyte ceramics: a brief review. Solid State Ion..

[41] Wang H (2014). Semiconductor heterojunction photocatalysts: design, construction, and photocatalytic performances. Chem. Soc. Rev..

[42] Lund PD (2017). Standardized procedures important for improving single-component ceramic fuel cell technology. ACS Energy Lett..

[43] Zhang Z (2010). Electrospun nanofibers of p-type NiO/n-type ZnO heterojunctions with enhanced photocatalytic activity. ACS Appl. Mater. Interfaces.

[44] Baliga, B. J. *Power Semiconductor Devices (General Engineering).* (PWS Pub. Co., 1995).

[45] Li Caixia, Dong Shihua, Tang Rui, Ge Xiaoli, Zhang Zhiwei, Wang Chengxiang, Lu Yupeng, Yin Longwei (2018). Heteroatomic interface engineering in MOF-derived carbon heterostructures with built-in electric-field effects for high performance Al-ion batteries. Energy & Environmental Science.

[46] Choi S (2018). Exceptional power density and stability at intermediate temperatures in protonic ceramic fuel cells. Nat. Energy.

[47] An H (2018). A 5×5 cm^2^ protonic ceramic fuel cell with a power density of 1.3 W cm^-2^ at 600 °C. Nat. Energy.

