# Supplementary material for: Shaping triple-conducting semiconductor BaCo0.4Fe0.4Zr0.1Y0.1O3-δ into an electrolyte for low-temperature solid oxide fuel cells
Source: Nat Commun. 2019 Apr 12;10:1707. doi: 10.1038/s41467-019-09532-z (PMC6461657; doi:10.1038/s41467-019-09532-z)
Supplement: Supplementary file 1 — Supplementary Info [file 41467_2019_9532_MOESM1_ESM.pdf]

Supplementary Information

Shaping triple-conducting semiconductor $BaCo_{0.4}Fe_{0.4}Zr_{0.1}Y_{0.1}O_{3-\delta}$ into an electrolyte for low-temperature solid oxide fuel cells

Chen Xia[1,3,], Youquan Mi[1], Baoyuan Wang[1,*], Bin Lin[4], Gang Chen[5], Bin Zhu[1,2,6,*]

[1]*Key Laboratory of Ferro & Piezoelectric Materials and Devices of Hubei Province, Faculty of Physics and Electronic Science, Hubei University, Wuhan, Hubei 430062, P.R. China*

[2]*Engineering Research Center of Nano-Geo Materials of Ministry of Education, Faculty of Materials Science and Chemistry, China University of Geosciences, 388 Lumo Road, Wuhan 430074, China*

[3]*Department of Energy Technology, KTH Royal Institute of Technology, Stockholm, SE-10044, Sweden*

[4]*School of Materials and Energy, University of Electronic Science and Technology of China, Chengdu 611731, PR China*

[5]*Liaoning Key Laboratory for Metallurgical Sensor and Technology, Northeastern University, Shenyang, 110819, China*

[6]*Department of Aero & Auto Engineering, Loughborough University, Ashby Road, Loughborough, UK, LE11 3TU*

*Corresponding authors:

Zhu (zhubin@hubu.edu.cn; J.Kim@lboro.ac.uk)

B. Wang (baoyuanw@163.com)

Supplementary Notes

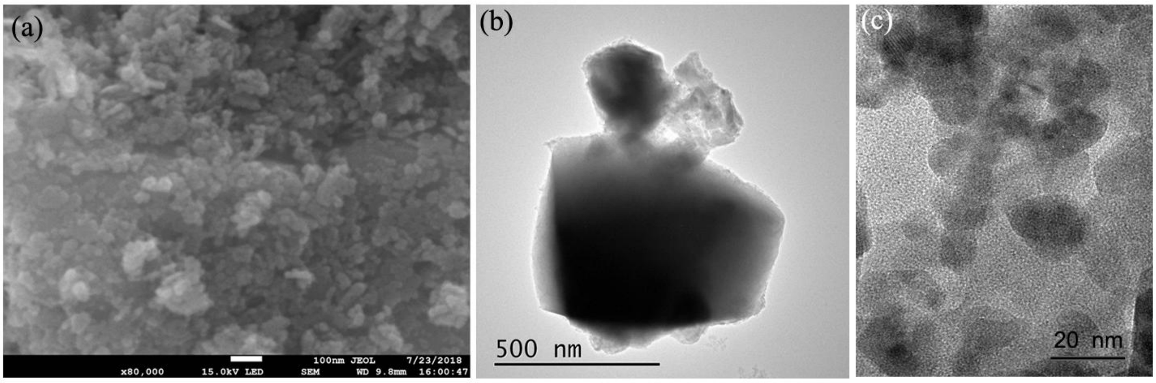

Supplementary Figure 1. (a) SEM image of the prepared BCFZY-ZnO composite, indicating that the particles are uniform and compact distributed; (b)(c) TEM images showing the interfaces formed between particles and grains.

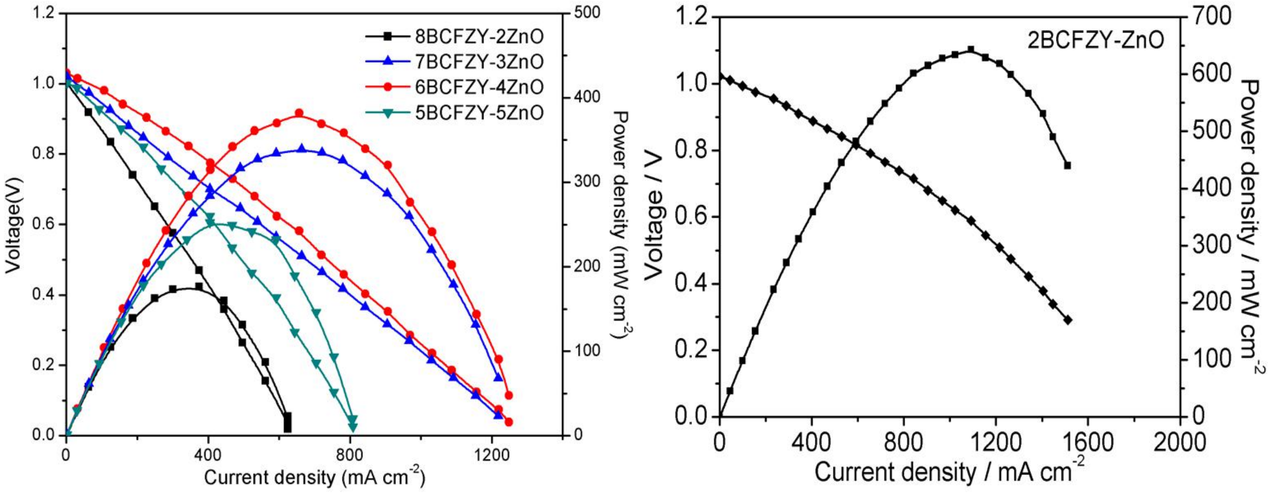

Supplementary Figure 2. Electrochemical performance of SOFC with 8BCFZY-2ZnO, 7BCFZY-3ZnO, 6BCFZY-4ZnO, 5BCFZY-5ZnO, and 2BCFZY-ZnO tested at 500 °C.

SOFC measurements were carried out with various mass ratios (8:2, 7:3, 6:4, 5:5) of BCFZY:ZnO in the composite to verify the optimal composition. Our investigation observed that the current density/power density were correlated with the mass ratios of BCFZY:ZnO. Better results are achieved at 7:3 and 6:4. Further test confirmed that 2BCFZY-1ZnO, which is between 7:3 and 6:4, was the optimal study case in our work, as shown above.

The findings also showed that all these mass ratios were sufficient to prevent the electrical percolation of BCFZY and ZnO particles, and reached almost the same OCVs (8:2, 7:3, 2:1, 6:4, 5:5 corresponding to OCV of 1.0 V, 1.02 V, 1.01 V, 1.03 V, 1.0 V, respectively). For the sake of best power density and high OCV, we confirmed the optimal mass ratio of 2:1 as our study case.

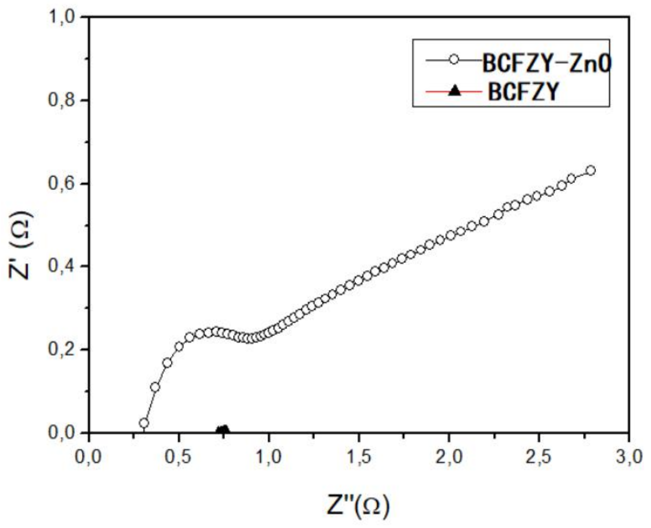

Supplementary Figure 3. Impedance spectra plots of the as-synthesized BCFZY and BCFZY-ZnO composite acquired at 500 °C in air. The EIS curve of BCFZY presents a spot in the real Z-axis, which should be due to the dominating intrinsic electron-hole conduction in air, while the EIS of BCFZY-ZnO displays an ionic behavior with

intercept at high frequency region, semicircle at intermediate frequencies and arc at low frequencies. This implies that the BCFZY-ZnO composite gained remarkably enhanced ionic conductivity after the introduction of ion-conducting ZnO.

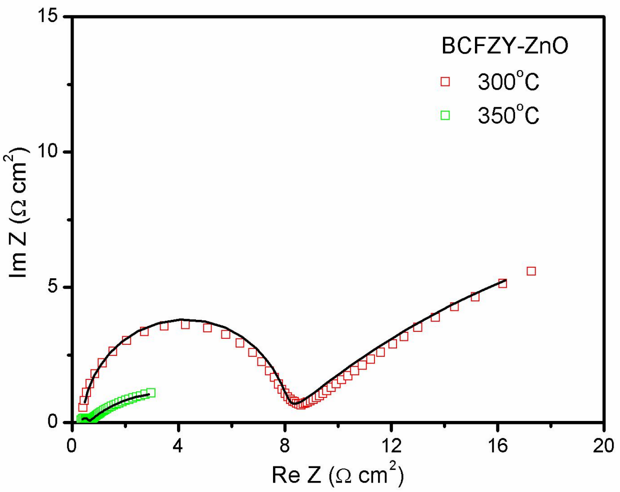

Supplementary Figure 4. EIS plots of BCFZY-ZnO composite acquired at 300 and 350 °C in H_2/air. Comparatively, the EIS curves at 450-550 °C represent smaller interceptions on Re Z-axis in the high frequency region and obviously minished semicircles at intermediate and low frequencies compared to the EIS at 350-400 °C.

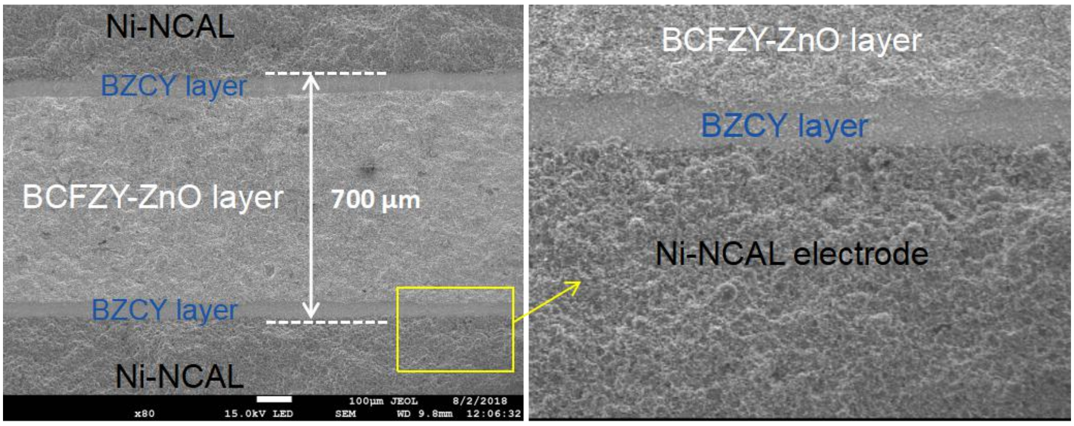

Supplementary Figure 5. Enlarged SEM images for the cross-sectional view of the O^{2-}/e^- blocking cell NCAL-Ni/BZCY/BZFCY-ZnO/BZCY/NCAL-Ni. Five layers corresponding to anode, BZCY filters, BZFCY-ZnO, and cathode are clearly distinguished in the SEM image, indicative of the successful construction of O^{2-}/e^- blocking cell. It can be observed that the BZCY/BCFZY-ZnO/BZCY trilayer is dense with a thickness of 700 μm.

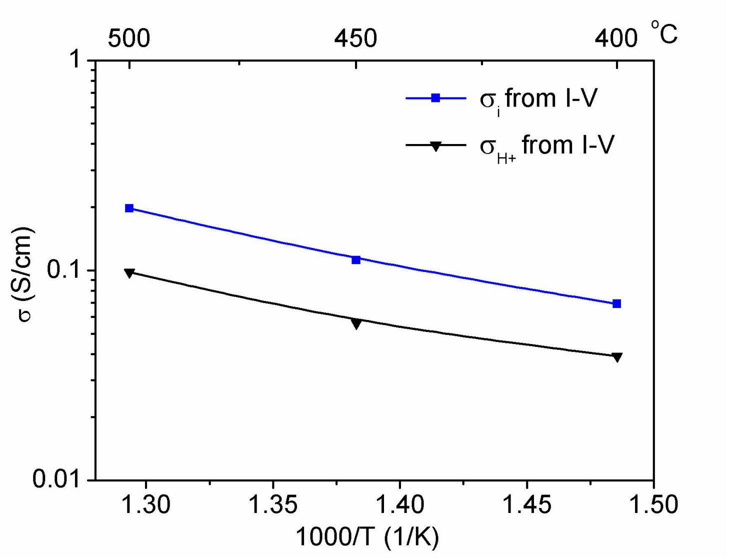

Supplementary Figure 6. Total ionic conductivity (0.07-0.20 S cm^{-1}) and specific proton conductivity (0.039-0.098 S cm^{-1}) of BCFZY-ZnO composite obtained from I-

V characteristics at 400 to 500 °C for comparison. The proton conductivity is almost half of the total ionic conductivity.

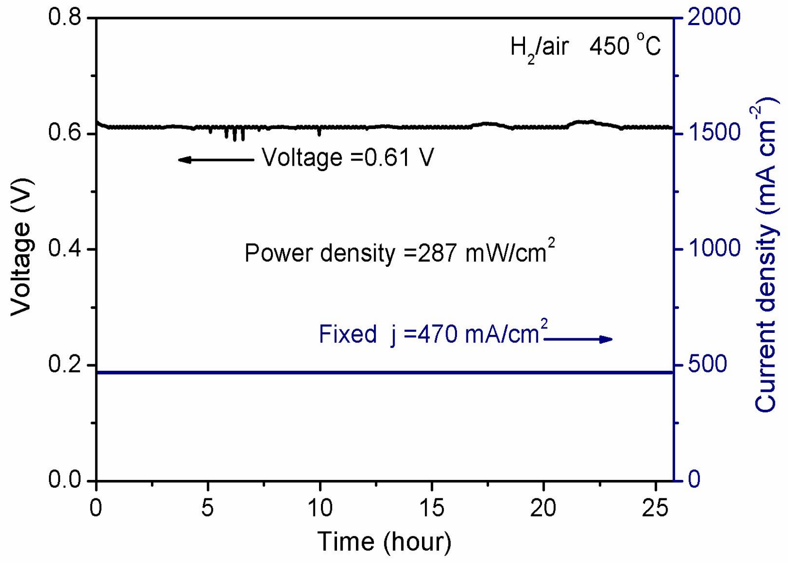

Supplementary Figure 7. Stability measurement of BCFZY-ZnO SOFC at a fixed current density of 470 mA cm^{-2} at 450 °C for 26 hours. A constant working voltage of 0.61 V was retained, which corresponding to the power density of 287 mW cm^{-2}. The cell voltage started to deviates from the initial value for the prolonged time of over 26 h, which should be by limited by the used fuel cell testing fixture and NCAL electrode.

Supplementary Table 1. The fitting parameters extracted from ZSimpWin with a equivalent circuit of $R_o(R_1Q_1)(R_2Q_2)$, where R represents a resistance, and Q is the constant phase element (CPE) representing a non-ideal capacitor. The R and Q have the units of Ω cm^2 and F cm^{-2}, respectively.

Sample	T (°C)	R_o	R_1	Q_1	n	C_1	R_2	Q_2	n	C_2
BCFZY-ZnO composite	500	0.1565	0.1088	7.28E-5	0.8379	8.162E-8	0.3647	2.076E-2	0.4337	1.32E-5
	450	0.2367	0.2785	3.33E-5	0.8001	5.127E-7	1.2604	4.853E-2	0.800	3.04E-2
	400	0.3960	0.2233	2.64E-4	0.5449	1.187E-6	2.0402	1.771E-4	0.9466	2.32E-4

The corresponding characteristic capacitance (C_i) for each process are calculated by : $C_i = \frac{(R_i Q)^{1/n}}{R_i}$. According to the obtaine C_i, the semicircles denoted by (R_1Q_1) and (R_2Q_2) can be ascribed to the grain-boundary and electrode polarization processes, respectively.

Supplementary Table 2. The obtained hall coefficient, hall mobility, carrier type, and carrier concentration parameters of BCFZY that treated in air and H_2 (500 °C), and ZnO treated in H_2 (500 °C) by the Hall-effect measurement.

Sample	Hall coefficient (cm^3 C^{-1})	Hall mobility (cm^2 $V^{-1}S^{-1}$)	Carrier type	Carrier concentration (cm^{-3})	Sheet Carrier concentration [m^{-2}]	Sheet hall coefficient [m^2 C^{-1}]	Resistivity [Ω m^{-1}]	Sheet resistivity [Ω sq^{-1}]	Hall voltage [v]
BCFZY in air	0.621	2.43×10^{-3}	P	1.01×10^{17}	1.19×10^{14}	5.62×10^{4}	2.56×10^{4}	2.17×10^{7}	1.05×10^{-3}
BCFZY in H_2	0.799	1.06×10^{-2}	N	7.81×10^{16}	5.08×10^{13}	1.23×10^{5}	7.56×10^{3}	1.16×10^{7}	-6.2×10^{-3}
ZnO in H_2	4.82×10^{-2}	1.07×10^{-3}	N	1.29×10^{16}	6.73×10^{12}	9.27×10^{5}	4.5×10^{5}	8.66×10^{8}	-2.5×10^{-3}

The two BZCFY samples were treated in air and H_2 at 500 °C, respectively, while ZnO sample was placed in H_2 at 500 °C. All these samples were treated for 1.5 h, followed by cooling down in N_2 atmosphere prior to Hall-effect measurements. It is indicated that the major charge carriers in the "BCFZY in air" sample was the hole, and in"BCFZY in H_2" and "ZnO in H_2" samples were electrons, suggesting that BZFCY is p-type semiconductor in air and n-type in reducing condition, while the ZnO maintains its n-type conduction feature in reducing condition. In fuel cell H_2/air operational condition, the p-type BCFZY and n-type ZnO can form p-n junction.
